# Study of Oseltamivir and Zanamivir Resistance-Related Mutations in Influenza Viruses Isolated from Wild Mallards in Sweden

**DOI:** 10.1371/journal.pone.0089306

**Published:** 2014-02-18

**Authors:** Goran Orozovic, Kanita Orozovic, Josef D. Järhult, Björn Olsen

**Affiliations:** 1 Section of Infectious Diseases, Department of Medical Sciences, Uppsala University, Uppsala, Sweden; 2 Department of Plant Biology and Forest Genetics Swedish University of Agricultural Sciences, Uppsala, Sweden; University of Georgia, United States of America

## Abstract

Resistance to neuraminidase inhibitors (NAIs) is a growing problem in battle against influenza A virus. However, little is known about the resistance of viruses isolated from dabbling ducks, the natural reservoir of the influenza virus. To our knowledge, no low-pathogenic avian influenza (LPAI) virus resistant to NAIs has been detected. The aim of this study was to investigate mallard isolates of influenza A virus previously identified to carry oseltamivir carboxylate (OC) or zanamivir (ZA) resistance-related mutations. In this work, 21 viruses belonging to the N1, N3, N6 and N9 subtypes were analyzed using a colorimetric NA inhibition assay. The results of assay showed no NAIs-resistant phenotype for any of the viruses. The R118K mutation was the most recurrent, as it was observed in all subtypes except for N6. IC_50_ values confirmed the differences in sensitivity to OC or ZA observed in the N1 and N2 groups of NAs. Furthermore, both wild types (WTs) in the N6 and one WT in the N9 subtype were less sensitive to ZA than were genotypically related mutants with R152K and R118K change in the respective subtypes. This may indicate that these and probably even other NAIs resistance-related mutations found in our virus collection were not induced by NAIs residuals in the environment and that the impact of such mutations in an avian influenza could be dependent on subtype, strain and host species.

## Introduction

The recent influenza A (H1N1) pandemic has once again confirmed that the influenza A virus has the potential to evolve into strains that can infect the global human population [1]. Such development implies that the new viral strain has gained changes that lead to antigenic drift in one or both of two major antigens, hemagglutinin (HA) and neuraminidase (NA). Alternatively, the virus may obtain a completely novel version of one of these proteins (antigenic shift). Independent of origin, these changes prolong the time required for the immune defense of the host to respond in an appropriate way [2].

There are 16 HA and 9 NA subtype versions circulating in nature [3] and each influenza A virus is named according to the subtype of HA and NA that it contains (e.g. H1N1, H6N5, or H7N2). Wild birds of the waterfowl group and, in particular, dabbling ducks such as the mallard (*Anas platyrhynchos*) are the natural reservoir of the majority of influenza A virus [4].

In a pandemic scenario, the use of two licensed neuraminidase inhibitors (NAIs) against influenza A, oseltamivir (Tamiflu) and zanamivir (Relenza), is crucial to the protection of the human population [5], [6], [7], [8]. Oseltamivir is administered as a prodrug, oseltamivir phosphate (OP) that is readily absorbed from the gastrointestinal tract. OP is rapidly converted to the active substance oseltamivir carboxylate (OC), primarily by esterases in the liver. Studies prior to 2007 have shown that human influenza viral isolates with resistance to OC were more common in Japan (2.2%) than in Europe (<1%) [9], [10]. However, during the following season, the average prevalence of OC-resistant H1N1 mutants in Europe grew to 20% [10]. This trend was observed worldwide and especially in U.S. at the beginning of 2009, giving rise to public health concerns about such developments [11], [12], [13], [14], [15]. In the season 2008/2009 the majority of worldwide circulating H1N1 viruses were resistant to OC [15].

When the environmental fate of OC is taken into consideration, the issue of NAI resistance becomes even more complex [16], [17], [18]. OC is degraded poorly in sewage treatment plants and surface water. It can be detected in aquatic environments where wild ducks may be exposed to the substance [18], [19], [20]. Low levels of OC in the sole water source of LPAI-infected mallards leads to resistance development [21], [22]. If NAI-resistant viruses establish themselves in the naturally circulating avian influenza pool, there is a risk of such viral pools giving rise to a human pandemic [2], [23]. Two of pandemic strains (H2N2, H3N2) in the previous century resulted from a reassortment between human and avian influenza A strains [24] while the first influenza pandemic of this century involving H1N1/09 virus was a result of multiple reassortments between swine, avian and human virus strains [25].

In our recent work, we demonstrated that OC and zanamivir (ZA) resistance-related mutations exist in viruses isolated from both wild and domestic birds, as well as in viruses isolated from swine and the environment [26]. Mutations associated with NAI resistance were identified in 15 out of 230 viral isolates from mallards that had been sampled at the Ottenby Bird Observatory (Öland, Sweden) during the period 2002–2008 [26]. In this work, 12 of these mutants and two mutants with mutations unrelated to NAI resistance have been analyzed by the colorimetric NA inhibition assay.

## Methods

### Ethics Statement

Ethical approval for trapping, sampling and keeping of birds was obtained from the Malmö/Lund Animal Research Ethics Board (M139-03).

### Virus sampling, detection and characterization

Cloacal samples from 230 mallard ducks (*A. platyrhynchos*) were collected as a part of the ongoing surveillance at the Ottenby Bird Observatory, Öland, Sweden. Further sample procedures, such as q-PCR, viral culturing in eggs and HA subtyping, have been described elsewhere [26], [27].

NA subtyping was performed by sequencing according to either Hoffman [28] (110 samples) or Orozovic [29] (120 samples) protocols. In the following steps, viral RNA isolation, RT-PCR, agarose gel electrophoresis and gel extraction were performed. Subsequently, the purified PCR products were sent to Macrogen (Seoul, Korea) for sequencing, after which the two sequencing reads were assembled. The obtained contigs were analyzed by BLAST and NA was subtyped [29].

### Genotype analysis

The sequence analysis was performed according Orozovic et al. [26]. Here a shortened description of the procedure is provided.

**Figure 1:**
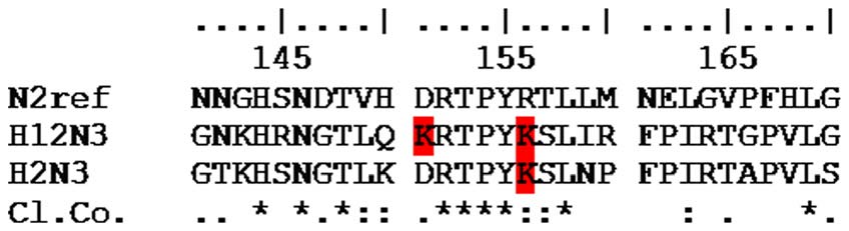
Sequence alignment of N2 reference, and three N1 sensitive mutants. The sequence interval between the 121^th^ and 149^th^ amino acid (marked with - - - ; N2 reference numbering) is excluded from alignment for practical reasons. The relevant mutation sites are indicated in red. (r) - reference sequence; Number within the parenthesis indicate code for virus isolates according Table 2. (3) - H1N1 R118K mutant; (4) - H1N1 R118K/D151N mutant; (14) - H6N1 R118K/D151N/D198N mutant. Cl.Co- ClustalW consensus sequence, “*” is used to indicate identical residues, “:” is used to indicate conserved substitution, “.” is used to indicate semi-conserved substitution, “ ” (empty space) is used to indicate dissimilar residues.

NAs of all subtypes have 19 conserved amino acids that function either as catalytic residues (R118, D151, R152, R224, E276, R292, R371 and Y406) or as framework residues (E119, R156, W178, S179, D198, I222, E227, H274, E277, N294 and E425) [26]. The majority of the mutations related to the NAI resistance have been found in or in close proximity to these sites. The literature describing NAI resistance mutations has been reviewed [30], [31], [32], [33], [34], whereupon nine OC (V116A, I117V, E119V, D198N, I222V, H274Y, R292K, N294S and I314V) and ten ZA (V116A, R118K, E119G/A/D, Q136K, D151E/G/N, R152K, R224K, E276D, R292K and R371K) NAI resistance-related mutations were identified (N2 numbering). ClustalW alignments of all sequences and the N2 reference sequence (GenBank ID: CAD35677) were performed for each NA subtype and the sequences were scanned (Figure 1) for each of the above mentioned mutations [26].

### Phenotype analysis

#### Colorimetric NA assay (CM)

The CM was performed according to the protocol developed by Warren [35], with the following changes. The volume of each solution in the assay was reduced five times compared to the original protocol in order to adapt the protocol to a 1.5 ml reaction tube [36], [37]. The final volume in the last step of the reaction was 1,400 µl.

A standard solution of sialic acid (SA; Sigma-Aldrich Corp.St. Louis, MO, USA) was prepared according to the original protocol to a final stock solution of 100 nmol/µl. Next, 20 µl of fetuin (Sigma-Aldrich Corp.St. Louis, MO, USA) substrate was added to 20 µl of viral solution, after which the tubes were incubated in a water bath at 37°C for 30 minutes. After cooling for 2 minutes at room temperature, 30 µl of periodate reagent was added to each reaction tube, whereupon the tubes were incubated at room temperature for 20 minutes. 200 µl of arsenite reagent was added to the tubes, which were subsequently spun and 500 µl of thiobarbituric acid reagent was added. The samples were incubated in a hot water bath for 15 minutes and cooled on ice immediately. To extract the colored product of the reaction, 600 µl of Warren's reagent was added to the reaction tubes. The tubes were then centrifuged at 4,600 rpm (app. 2,000 RCF). Finally, 300 µl of the upper phase was pipetted in flat bottom plates and the OD was measured at 549 nm on a FLUOstar Optima instrument (BMG Labtech GmbH, Ortenberg/Germany).

All isolates were analyzed in triplicates using a dilution series of 0.5 log10. The virus isolates were titered and the OD signals in range between 0.2 and 0.9 at 549 nm (3–20 nmol of SA) resulted in a linear function. The volume of allantoic fluid that gave signal of approximately 15 nmol of SA was used in the inhibition assay.

#### Colorimetric NA inhibition assay (CMI)

The NAIs OC (Hoffmann-La Roche, Basel, Switzerland) and ZA from a single dose of Relenza (GlaxoSmithKline, Uxbridge, United Kingdom) were employed in the assay. The concentrations of both inhibitors were calculated using a 0.5 log10 dilution series (e.g. 0.000 nM, 0.032 nM, 0.10 nM etc.). Next, 20 µl of each inhibitor was added to the viral solution prior to the addition of the substrate, whereupon the mixture was incubated in a water bath at 37°C for 30 minutes. Thereafter, the protocol was followed as described above for the CM.

For each NA subtype, two wild types (WT) of the same subtype that lacked NAI-related mutations were employed. All analyzed isolates were run in triplicate and IC_50_ values were calculated using nonlinear regression and dose-response equation in GraphPad Prism version 5.00 for Windows (GraphPad Software, San Diego California USA, www.graphpad.com). The criteria for resistance were adopted from Mishin et al. [38]. According this work the virus is regarded as resistant if it exhibits IC_50_ of ≥15 nM and 8.0-fold higher than IC_50_ of the WT. The IC_50_ values of sensitive and resistant viruses were retrieved from the available published literature [31], [32], [34], [38], [39], [40], [41]. The variations in IC_50_ values due to use of different NA inhibition assays i.e. chemiluminescent (CL), fluorescent (FL) and CM were ignored since they do not influence the conclusions on virus susceptibility to NAIs [37].

### Statistical analysis

A one-way ANOVA with a post-hoc Tukey test was performed to test for differences among IC_50_ values. A paired t-test was performed to analyze the influence of separate mutations on the enzyme performance in the assay with respect to each inhibitor. All tests were performed using GraphPad Prism.

## Results

### Genotype analysis

A detailed genotype analysis of the mutant sequences was previously reported by Orozovic et al. [26] and a summary of these results is presented in Table 1. In this work, two additional sequences that carried resistance-unrelated mutations were identified. One isolate (H8N1) with a D151K mutation and one isolate (H2N3) with R156K mutation were revealed.

**Table 1:** Distribution of mutations with regards to NA subtype.

NA	No. of isolates (x)	Mutation	No. of mutants (y)	% (y/x)
N1		R118K	1	2.6
		R118K D151N	1	2.6
		R118K D151N D198N	1 (3) 1)	2.6 (7.7)4)
		D151K	1 2)	2.6
		I222V	1	2.6
All	39		5	12,8
N2		-	0	0.0
All	77		0	0.0
N3		R118K	1 3)	4.4
		R156K	1 2)	8.7
All	23		3	13.0
N5		R118K R152K D198N	1 3)	8.3
All	12		1	8.3
N6		R152K	6 (2) 3)	10.9
All	55		6	10.9
N8		-	0	0.0
All	13		0	0.0
N9		R118K	2	13.4
		R118K D151N	1	7.7
All	11		3	27.3
Total	230		18 (15) 4)	7.4 (6.5) 4)

Mainly adopted from Orozovic et al. [26].

1) Sum of all R118K mutations.

2) Mutation unrelated to inhibitor resistance.

3) Isolates that were not analyzed.

4) Previously published values.

### Phenotype analysis

#### Inhibition of WTs by NAIs

**Figure 2:**
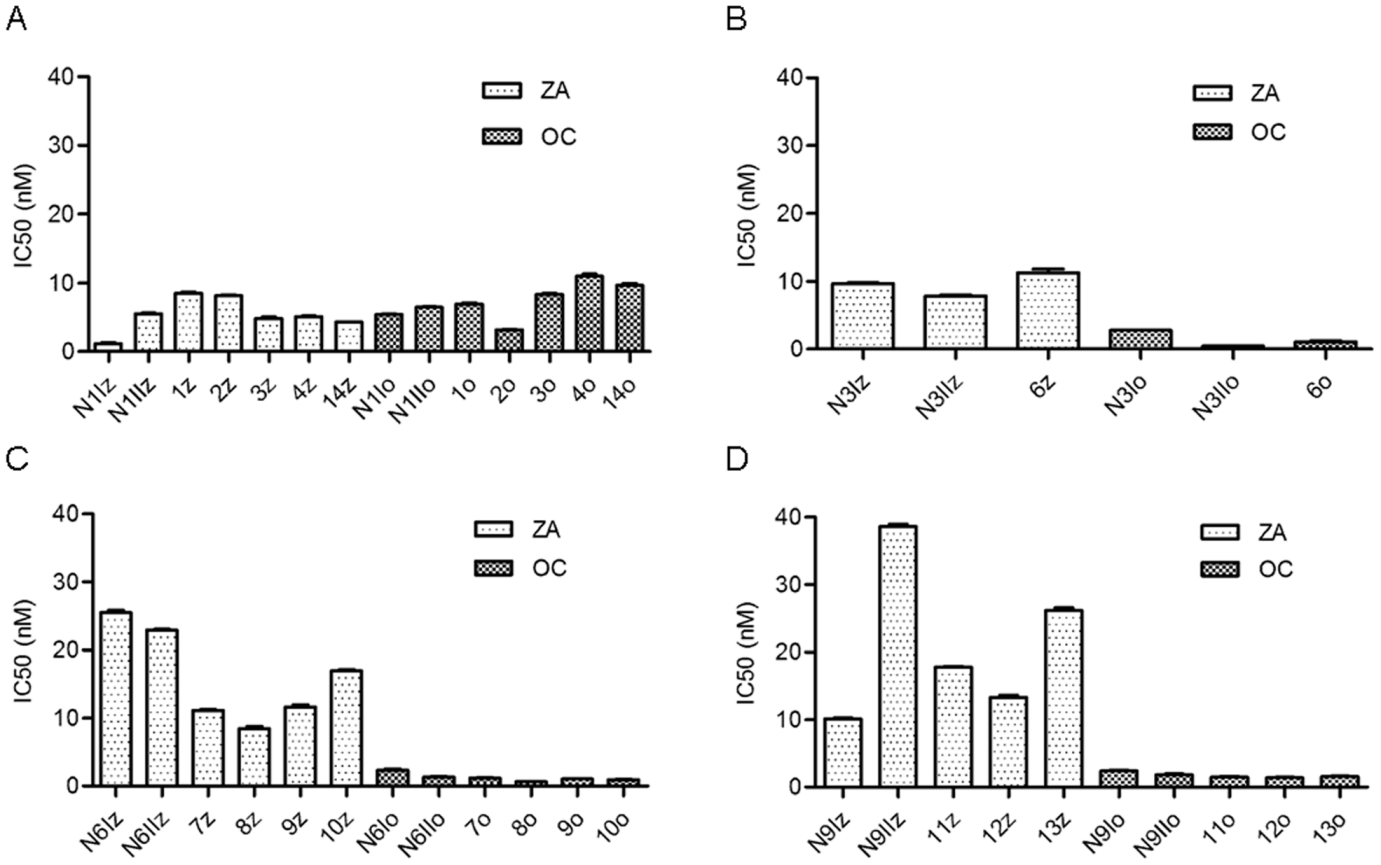
IC_50_ values of viral isolates analyzed by CM. Each bar shows mean ± SE IC_50_ of three replicates. The x-axis shows the viral isolates. NxI and NxII represent wild types (WTs) and 1–14 represent mutants. For codes of enumeration, see Table 2. z – inhibition by ZA; o – inhibition by OC. On the y-axis, the IC_50_ values are expressed as concentration of inhibitor in nM. All means were tested by one-way ANOVA and post-hoc Tukey tests. A) N1 subtype; B) N3 subtype; C) N6 subtype and D) N9 subtype.

Data analysis showed a varying pattern of IC_50_ values depending on which inhibitor was employed and which viral subtype was analyzed. Generally, all IC_50_ values fell in the range from 0.4 to 38.6 nM (Figure 2A–D and Table 2). After inhibition by ZA, the highest IC_50_ value (38.6 nM) was found in the N9_II_ WT, while the lowest IC_50_ value (1.2 nM) belonged to the N1_I_ WT.

**Table 2:** Performance of virus isolates in CM assay.

Code 1)	Virus subtype	Mutation	Type of residue 3)	IC_50_ ± SE (nM)	
				ZA	OC
N1_I_	H1N1	no	WT	1.2±0.2	5.4±0.0
N1_II_	H1N1	no	WT	5.5±0.1	6.4±0.1
1*	H1N1	I222V	F	8.5±0.3	6.9±0.2
2	H8N1	D151K 2)	C	8.2±0.1	3.2±0.1
3	H1N1	R118K	C	4.9±0.2	8.4±0.1
4	H1N1	R118K	C	5.1±0.2	11.0±0.3
		D151N	C		
14	H6N1	R118K	C	4.3±0.1	9.7±0.3
		D151N	C		
*		D198N	F		
N3_I_	H2N3	no	WT	9.7±0.1	2.8±0.0
N3_II_	H5N3	no	WT	7.9±0.2	0.4±0.0
6	H2N3	R156K 2)	F	11.3±0.5	1.1±0.2
N6_I_	H4N6	no	WT	25.4±0.6	2.4±0.2
N6_II_	H4N6	no	WT	22.9±0.2	1.3±0.1
7	H4N6	R152K	C	11.1±0,1	1.2±0.1
8	H4N6	R152K	C	8.4±0.4	0.6±0.1
9	H4N6	R152K	C	11.7±0.3	1.1±0.0
10	H11N6	R152K	C	17.0±0.2	0.9±0.1
N9_I_	H11N9	no	WT	10.1±0.2	2.4±0.1
N9_II_	H11N9	no	WT	38.6±0.3	1.8±0.1
11	H11N9	R118K	C	17.8±0.1	1.5±0.2
12	H11N9	R118K	C	13.3±0.3	1.4±0.1
13	H11N9	R118K	C	26.1±0.4	1.6±0.1
		D151N	C		

1) Code for virus subtypes; *- mutations induced by OC (adopted from literature); without *- mutations induced by ZA (adopted from literature).

2) Mutation unrelated to inhibitor resistance.

3) F - framework residue; C - catalytic residue; WT- wild type.

After inhibition employing ZA the IC_50_ for WTs differed more markedly for subtypes N1 and N9 (Figure 2A and 2D) than for subtypes N3 and N6 (Figure 2B and 2C).

Conversely, the WTs following OC inhibition were less variable (Figure 2A–D), with the exceptions of the N3_I_ and N3_II_ isolates, in which IC_50_ values of 2.8 nM and 0.4 nM (seven times difference), respectively, were detected. Differences between other pairs of IC_50_values for WTs varied approximately 2.0-fold (Table 2).

An additional paired t-test analysis investigating the inhibitory effects of NAIs on each WT isolate was performed. In summary, the WTs of the N1 subtype were more sensitive to ZA than to OC, while the opposite trend was observed in subtypes N3, N6 and N9 (Figure 2A–D).

#### Inhibition of the mutants by NAIs

All IC_50_ values of mutants in the N1 subtype following inhibition by ZA were higher than the corresponding mean value of the N1_I_ WT (Figure 2A; Table 2). The situation was more complex when N1 mutants were compared with the N1_II_ WT (Figure 2A; Table 2). Three mutants (3z, 4z and 14z) had lower IC_50_ while the rest of mutants (1z and 2z) had IC_50_ higher than IC_50_ of that WT. The H2N3 mutant (Figure 2B: 6z; Table 2) had a mean IC_50_ slightly higher than the IC_50_ values of either WT (Figure 2B). In the N6 subtype, IC_50_ values of all mutants were lower than IC_50_ values of the WTs (Figure 2C; Table 2). Two contradictory IC_50_ patterns between the WTs and the mutants were observed in the N9 subtype (Figure 2D; Table 2) since the values of IC_50_ in the WTs differed by a 3.8-fold. Compared to WT N9_I_ all mutants were less sensitive to NAIs while in comparison with WT N9_II_ all mutants were more sensitive to NAIs.

The IC_50_ values in the N1 subtype following inhibition by OC showed a different pattern compared to inhibition by ZA (Figure 2A; Table 2). In this case three mutants with R118K change (3z, 4z and 14z) and one mutant with I222V change had IC_50_s higher than corresponding IC_50_s of both WTs while only mutant with D151K change had IC_50_ lower than IC_50_s of WTs. The H2N3 viral isolate (Figure 2B: 6o) had a higher IC_50_ than that of N3_II_ WT and a lower IC_50_ than that of N3_I_. In relation to WTs, the IC_50_ pattern for both N6 and N9 subtypes following inhibition by OC was more uniform than the pattern seen following inhibition by ZA (Figure 2C and 2D; Table 2). In both cases IC_50_s of all mutants in both NA subtypes were lower than corresponding IC_50_ values of WTs.

The majority of the pairwise comparisons performed using one-way ANOVA followed by post-hoc Tukey test and including inhibition with both NAIs revealed significant differences (p<0.05 and lower) between the IC_50_ values within each NA subtype (Figure 2A–D; Table 2). The most similar IC_50_ values were found with OC inhibition among mutants in the N6 and N9 subtypes (Figure 2C and 2D). The paired t-test confirmed that both WTs and mutants had different sensitivities to the two NAIs.

#### Effect of the R118K mutation on different subtypes

The mutation R118K did not influence NAI sensitivity to any of inhibitors in either the N1 or the N9 subtypes. The obtained IC_50_ values of the N1 and N9 subtypes was consistent with the separation pattern of the N1 (N1 subtype) and N2 (N9 subtype) groups of NAs (Figure 2A and 2D).

## Discussion

### Genotype analysis

In our work we defined WT of NAs for each subtype when its amino acid sequence lacked changes in residues at locations where previously published NAI resistance-related mutations have been found according to N2 subtype numbering. Because WT and mutant sequences differed in several amino acid residues at other locations of the NA gene we ignored a possible influence of these differences on the performance of NA in CMI. It has been hypothesized that the effect of these amino acids on the enzymatic activity of NA is not of such magnitude that it disqualifies the chosen WTs in their role as negative controls. In an attempt to scrutinize this hypothesis, we chose two virus isolates as WTs. That gave us the opportunity to see how inhibitor resistance-unrelated variations influence WTs. The observed difference in CMI performance between two WTs for every subtype after inhibition with either inhibitor (Figure 2, Table 2 and *Discussion*) most likely demonstrates a natural variation in NAI susceptibility that still lies well within the sensitive range. This is in accordance with previous observations of a large variation in NAI susceptibility in avian influenza viruses [40], [41]. Although we have only tested two WT isolates per subtype, the results are still coherent with the thought that non-resistance related mutations do not significantly change WT sensitivity to NAIs and that the WT isolates are suitable as negative controls in our study. The same hypothesis was even used in regards to NAs from different hosts and subtypes. Consequently our interpretation of results and derived conclusions are based on this rationale.

### Phenotype analysis

#### Inhibition of the WTs by NAIs

All NA subtypes could be divided in two groups according their phylogenetic origin. Group N1 includes the N1, N4, N5 and N8 subtypes whereas group N2 includes the N2, N3, N6, N7 and N9 subtypes [30], [42]. It has been reported that NAs from the N1 group were more sensitive to ZA than to OC and the opposite has been reported for NAs from the N2 group [30], [39], [41]. An explanation for these differences proposed in a publication by Russell et al. [42] was related to the presence of the 150 cavity found only in the N1 group. In addition to other residues, the cavity is formed by the 150 loop (amino acid residues 147–152) located above the active site. The division into the two NA groups with regards to the two NAIs was observed in all subtypes investigated in this study (Figure 2A–D), with the exception of two N1 mutants.

The sequence differences within each subtype and, in particular, between subtypes can influence the microenvironment in and around the active site as well as NA monomer interactions, the degree of NA glycosylation, the optimum pH of the enzyme etc. The interaction between HA and the substrate fetuin and the balance between HA and NA activities that may depend on the HA subtype and its variants could also influence NA catalysis [43].

The IC_50_ values of the majority of WTs after inhibition by either NAIs were ≤10 nM. Both WTs of N6 subtype and one WT of N9 subtype had IC_50_ of 22.9 and 25.4 nM for the first and 38.6 nM for the second subtype after inhibition by ZA (Table 2). Human viruses of H1N1 subtype, on the other hand, had much lower IC_50_ values for both NAIs. The analyses by CM assay showed that the IC_50_ values fell within the range of 2.4–7.0 nM. In the same work CM assay yielded IC_50_ values 1.4–5.0-fold (ZA) and 1.6–6.5-fold (OC) higher than the IC_50_ values yielded by FL assay [37]. Accordingly, it was possible to compare the IC_50_ values between WTs analyzed by FL and CM assays for avian viruses.

Depending on criteria used, IC_50_ values for all WTs in this study correspond well with IC_50_ values found in NAI-susceptible and/or NAI-moderately susceptible avian viruses [38], [40]. These values also fit very well with earlier work on NAIs inhibition of different avian influenza subtypes published by Govorkova et al. [41]. In that study virus isolates were collected from different parts of world, different time periods (newest isolates collected 1997) and different avian species. Such similarity in NA activity between the two studies could imply the functional conservation of NA enzymatic performance through time and space as well as its independence on possible differences in NA polypeptide sequences. In that case such NA functional conservation fits very well with the rationale used in our study.

#### Inhibition of the mutants by NAIs

The IC_50_ of NAI-resistant viruses or viruses with a reduced susceptibility to NAIs vary depending on mutation position, identity of the substituting amino acids, number of mutations in a single NA sequence (two or more), NA subtype, HA subtype, the host origin and choice of inhibitor and NA inhibition assay [31], [32], [34], [38], [40].

For example, the OC resistance-related mutation H274Y (N2 numbering) in pandemic H1N1 A/Osaka/180/2009 resulted in IC_50_ of 93.9, 1,048.2 and 1,333.8 nM analyzed by CL, FL or CM assay respectively [37]. In the case of H5N1 2.2 A/Turkey/15/06 recombinant virus and inhibition by OC the IC_50_ for E119A, H274Y and N294S were 236.5, 6308.0 and 424.2 nM respectively (FL assay). The inhibition by ZA of the same recombinant virus that has V116A or E119A mutation resulted in IC_50_ of 32.8 and 1253.8 nM respectively [32]. In order to reduce influence of the described factors in the estimation of resistance to NAIs the criteria for estimation of resistance published in Mishin et al. [38] was employed.

In our virus collection for all mutants the existing mutations did not alter NA susceptibility to either of the NAIs compared to the WTs. Hence, the mutants showed the same groupings into N1 and N2 divisions previously observed in WTs, with the exception of I222V and D151K mutants belonging to N1 subtype. Although these mutants belong to the N1 group, they had a higher IC_50_ following ZA inhibition compared to OC inhibition. In this case, it is possible that these mutations altered the sensitivity of the virus to the inhibitor and that this alteration affected group assignment.

In the N6 subtype, the R152K mutation appeared to increase the sensitivity to both inhibitors in all mutants compared to both WTs (Table 2). A similar effect was observed in the R118K mutants of the N9 subtype in comparison with one of the WTs. Such responses could indicate that these mutations were natural versions of the NA gene and not changes induced by the selective pressure posed by xenobiotics. The presence of additional mutations in double and triple mutants (N1 and N9 subtypes) did not contribute to any considerable differences in sensitivity to the inhibitors.

#### Effect of R118K mutation in different subtypes

The IC_50_ of the N1 and N9 subtypes followed the rules of separation in two NAs groups (Figure 2A and 2D). Previously, the R118K mutation had been introduced into the H3N2 A/Wuhan/359/95-like virus strain using reverse genetics and this mutant was able to proliferate only in the presence of exogenous (bacterial) NA [34]. In the background of the human virus, the recombinant virus could not be analyzed by FL assay, while the wild bird viruses of N1 (N1 group) and N9 (N2 group) subtypes investigated in this paper could be both propagated in eggs and analyzed by CM assay. The mutation R118K inhibited replication of the human strain but did not appear to affect the fitness (i.e. the replication capacity of the virus to produce fertile offspring in given environment) of the avian strain. It is possible that during viral adaptation to a new host, i.e. humans, the NA has lost certain properties that otherwise might make it possible for virus to retain fitness despite mutations in NA catalytic site, or it could be due to variation in the genetic background of different strains.

We stated above that the NAI resistance-related mutations in our virus collection were most likely not induced by NAIs in environment. However this possibility cannot be excluded completely and it may be applicable for all NAs having NAI related mutations. If this was the case it means that such NA versions gained additional mutations at other places in the gene leading to regaining the sensitivity to NAIs. That may be possible provided that the NAI resistance-related mutations decreased fitness and sensitivity of viruses and that the virus having NAs with additional mutations have been compensated for the loss of fitness as well as sensitivity to NAIs.

## Conclusions

NAI resistance-related mutations are found in the natural population of viruses that infect wild mallards but in our virus collection they do not significantly affect NA sensitivity against NA inhibitors. The presence of R118K mutation in N9, R152K in N6 and probably even majority of other mutations in N1 and N3 are rather due to natural variation of NA than induction by NAI. Such conclusion is based on the fact that these mutations could in many cases somewhat increase sensitivity to the both NAIs compared to WT viruses. This also suggests that genotypic changes in influenza A viruses must be interpreted in the context of virus subtype, host species and virus strain. Finally it is important to emphasize the importance of a continuing regular screening of LPAI viruses in wild birds for NAI resistance. The screening is important as influenza A viruses circulating in wild birds have a potential to gain new mutations or combinations of existing ones that can lead to antiviral resistant variants that may pose a risk to public health.
